# Evaluation of an Ileorectostomised Rat Model for Resistant Starch Determination

**DOI:** 10.3390/nu13010091

**Published:** 2020-12-30

**Authors:** Damien P. Belobrajdic, Anthony R. Bird

**Affiliations:** CSIRO Health and Biosecurity, Adelaide, SA 5000, Australia; tony.bird@csiro.au

**Keywords:** ileostomy, high amylose wheat, resistant starch, rat

## Abstract

The human ileostomy model, widely considered the benchmark for determining in vivo starch digestibility, has disadvantages. The ileorectostomised rat model (IRM) is a possible surrogate but evidence as to its validity is scant. In this preliminary study, the resistant starch (RS) content of test breads made from refined low (LAW-R) and high amylose wheat (HAW-R) flours was established in a randomised cross-over trial involving six human ileostomy participants. Starch digestibility of refined breads and diets made from these flours was then evaluated in ileorectostomised rats using a similar experimental format. Physical performance measures and other data were also collected for the rat model. The amount of RS in the low- and high-amylose breads as measured using the human model was 0.8 ± 0.1 and 6.5 ± 0.3 g/100 g, respectively. The RS level of HAW-R bread determined using ileorectostomised rats was 5.5 ± 0.8 g/100 g, about 15% less than that recorded in the human study, whereas for conventional wheat breads the models produced similar RS values. While offering promise, further validation using a wide variety of starchy food products is needed before the IRM can be considered an acceptable alternative for RS determination.

## 1. Introduction

Foods containing starches that undergo less small intestinal digestion have important implications for human health. By escaping digestion in the upper gut, starch that reaches the large bowel (resistant starch, RS) becomes a substrate for microbial fermentation and leads to higher production of short-chain fatty acids (SCFA). These acids are not only integral to bowel health and protection against DNA damage [1], but also influence metabolism of peripheral tissues, including skeletal muscle, adipose tissue depots and liver, and contribute to the regulation and strengthening of immune system function and responses to infection [2,3]. Additionally, slowing the rate and extent of amylolysis in the small bowel lowers the glycaemic response to food and improves blood glucose control [4], which can contribute to the prevention and management of type-2 diabetes [5,6].

Determination of the RS content of a food can be problematic given that starch digestibility is affected by the degree of food mastication and intestinal transit rate, which vary considerably between people of differing age and health status [7,8,9,10]. Currently, balance studies conducted in people with an ileostomy offer the most reliable means of directly determining the RS content of foods, subject to implementation of and adherence to strict volunteer recruitment criteria and study protocols [11]. However, the future feasibility of using this method for quantifying RS is limited by the availability of study participants who meet the study criteria because the surgical procedure (ileostomy) to establish a permanent abdominal stoma is becoming increasingly redundant. Whilst other approaches for investigating intestinal assimilation of foods have been developed, including intubation of the small intestine to permit sampling of ileal contents, their accuracy is questionable [12]. Small animal models, such as the ileorectomised rat model (IRM), are a possible alternative to the human ileostomy model. It has previously been used to study upper gut nutrient digestibility [13,14] and is a preferred model over terminal ileal sampling from conventional animals for technical and logistical reasons. Although measures of starch digestibility have been compared between the IRM and in vitro models [13,15], the validity of IRM for quantifying RS has not been established by direct comparison with the human ilesotomy model.

Western diets typically contain little RS because staple foods, such as cereals, are highly processed and contain starches that are readily digested in the upper gut. Consequently, RS intakes are commonly below half the level of 20 g of RS per day as suggested by Baghurst et al. [16] for providing a range of health benefits [16,17,18,19,20]. To help address this, we developed a wheat variety that contains high levels of amylose, a type of starch that has low digestibility compared to conventional wheat varieties [21,22]. Although bread made from this wheat variety has a lower glycaemic impact compared to conventional bread [23], its RS content has not been established.

The purpose of this study was to gauge the potential of the IRM for determining the RS content of foods and whether it might provide a viable alternative to the human ileostomy model for investigating small bowel starch digestibility. A further objective was to establish the RS content of refined and wholemeal breads made from a novel high amylose wheat (HAW). To do this, we directly quantified the physiological RS content of breads made from a conventional (low amylose) wheat and the novel HAW in ileorectostomised rats and healthy ileostomates. The ileorectostomised rats were also fed standard rodent diets made with refined flours from conventional or HAW to assess the usefulness of this model for measuring the RS level of flours in a dietary context.

## 2. Results

### 2.1. Human Study

#### 2.1.1. Compliance

The study participants were 100% compliant with consuming the entire serve of each test bread (64 ± 0.5 g) at the allocated time. All participants adhered closely to consuming the foods prescribed with only minor deviations recorded and calculations of starch intake were adjusted accordingly.

#### 2.1.2. Stoma Digesta Excretion and Starch Digestibility

The digesta total wet weight and moisture content for all study participants were in a healthy range (Table 1), similar to that reported in previous studies [11,24] indicating that they had a well-functioning digestive tract. All test breads showed similar stoma total wet digesta output. The dry matter content of the digesta was higher following wholemeal high amylose wheat (HAW-W) bread consumption compared to the wholemeal low amylose wheat (LAW-W) bread but did not differ between the breads made from the refined flours (Table 1).

Digesta starch output and digestibility are shown in Table 1. Even though starch intake was lower when study participants consumed breads made from HAW compared to LAW (HAW; 19.4 ± 2.6 g, LAW; 23.6 ± 3.0 g, *p* < 0.01), the HAW breads had greater starch output compared to LAW breads (Table 2). Subsequently, the starch from HAW breads (3.9 ± 0.5 g/100 g) was 9-fold less digestible than breads made from LAW (0.5 ± 0.1 g/100 g, *p* < 0.0001), irrespective of whether the breads were made from refined or wholemeal flour (Table 1).

### 2.2. Rat Ileostomy Study

The digesta total wet weight was higher when ileorectostomised rats consumed diets containing HAW bread or flour compared to diets containing LAW bread or flour (Table 2). This difference was due to a two-fold higher excretion of dry matter and not moisture content of the digesta which was similar between the dietary treatments.

Bread made from refined HAW flour had a higher quantity of indigestible starch compared to bread made from refined LAW flour (Table 2). The RS levels of the LAW and HAW breads were 0.76 ± 0.22 g/100 g and 5.48 ± 0.76 g/100 g, respectively. Diets made from refined HAW flour had a higher quantity of indigestible starch compared to diets made from refined LAW flour (Table 2). Bread had a higher quantity of indigestible starch compared to diets that contained flour either as LAW (flour 0.8 ± 0.4, bread 1.8 ± 0.5% indigested starch, *p* = 0.009) or HAW (11.2 ± 1.6, HAW bread 15.6 ± 2.0% indigested starch, *p* = 0.018).

**Figure 1:**
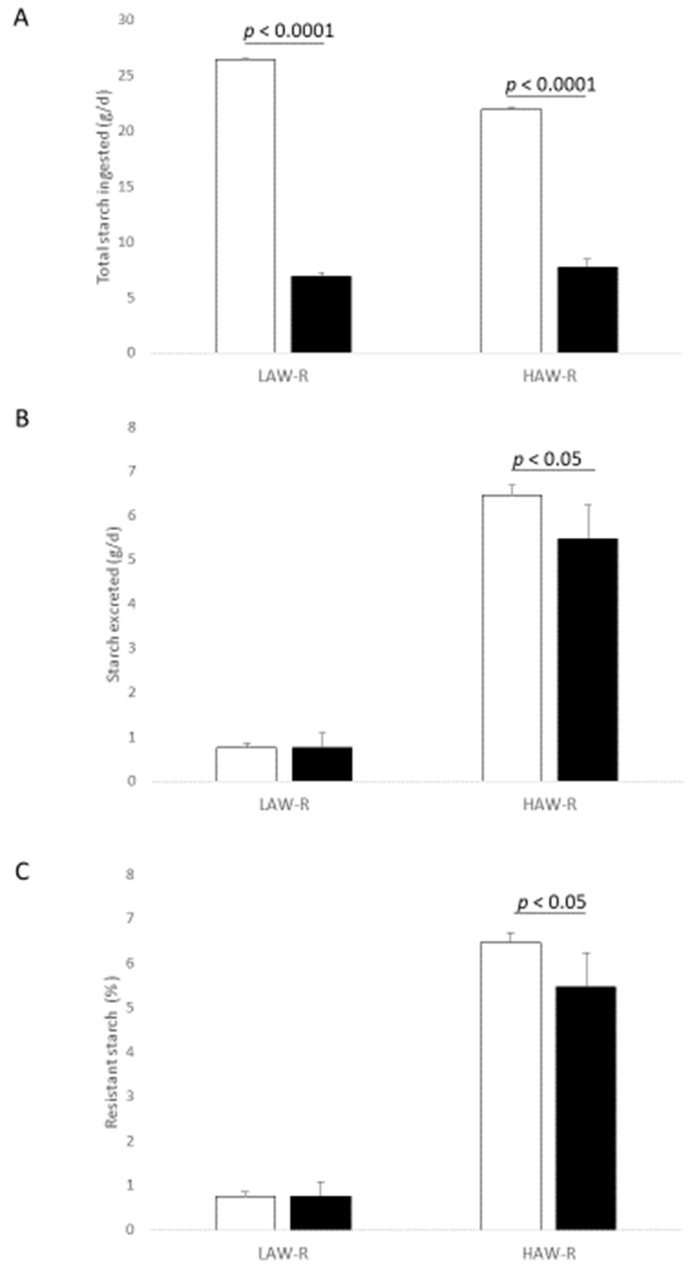
Comparison of (**A**) total starch ingested (**B**) starch excreted and (**C**) resistant starch content of breads made from refined flours in human ileostomates and ileorectomised rats. Values are means with standard deviation of the mean represented by vertical bars. □ Human ileostomate data *n* = 6, ■ ileorectomised rats data *n* = 3. *p* values were obtained by an unpaired *t*-tests.

Comparison of the two models showed that total starch intake was higher for human ileostomates compared to ileorectostomised rats which reflected the larger volume of food consumed by humans (Figure 1). The two models showed similar RS values for LAW-R bread but, for the HAW-R bread, the rat model yielded a value that was 15% lower than that obtained in the human trial (Figure 1).

During the initial 11 day phase of consuming the test diets the ileorectostomised rats fed diets containing HAW-R flour had lower body weight gain compared to diets containing LAW-R flour (*p* < 0.05) and a trend to a lower feed conversion efficiency ratio (*p* = 0.082) (Table 3. However, during the second phase (5 day) of the test diets and bread phase there was no difference in body weight gain between the dietary treatment groups (Table 3).

Rats consuming the LAW-R and HAW-R test diets and breads had similar feed intakes (Table 3).

## 3. Discussion

The current study further supports the ileostomy model as a direct and precise approach for determining the RS content of foods. We showed that there was minimal variation in the amount of starch escaping the terminal ileum, within and between volunteers. Functional variables (stomal output, dry and wet weight) were also consistent thereby providing further evidence of good participant compliance with the study protocol. Importantly, bread made from conventional wheat flour contained only small amounts of RS (0.6–0.8% RS). The low level of RS is consistent with previous studies which show that white bread and other bakery food products contain little starch (<2.5%) that escapes digestion [24,25,26]. Reported differences in the levels of RS measured in staple foods is likely due to differences in starch source, food physical structure/form, processing and storage, and analytical methodology.

In the present study we report for the first time in humans that HAW is a rich source of RS. We showed that bread made from wholemeal or refined HAW contains eight-fold more RS than breads made from conventional wheat (wholemeal or refined). Consequently, the calculated amount of RS delivered to the large bowel from a standard serve (1 × 40 g slice) of these breads is 2.6 g for refined HAW bread and 2.0 g for wholemeal HAW bread. The RS content of bread made from wholemeal HAW is slightly lower than bread made from refined HAW flour, due primarily to the bran component of the wholemeal flour, however, the overall level of fibre is greater. The markedly higher level of RS in HAW has considerable potential for improving metabolic and bowel health. We have previously shown it is effective in modulating glycaemic response. Substitution of conventional LAW flour with HAW flour lowered the postprandial glycaemic response of bread by 39% and the insulinemic response by 24%, and these changes were consistent with the lower circulating concentrations of incretin hormones [23]. A currently unpublished clinical study has shown that HAW improved measures of gut health [27]. This finding is supported by animal studies. In rats we have shown that HAW feeding resulted in reduced colonocyte DNA damage, increased large bowel production of SCFA and reduced pH and protein fermentation metabolites [28,29]. Commonly consumed foods in the typical western diet contain high levels of starch, such as bread and noodles. If these foods are made using HAW flour [22], this could be an effective means for people to easily double their RS consumption, achieve the recommended intake levels of RS (20 g/day), and improve gut and metabolic health [16].

In the current study we showed that starch digestibility in wholemeal and refined bread were similar. Although HAW-R had slightly higher levels of RS compared to HAW-W, this reflects the higher starch content of HAW-R, which is predominately amylose. This finding is also consistent with previous studies that showed that the degree to which whole grains are milled into flours (e.g., particle size) affects glycaemic control [30]. We have also shown that the breads made from HAW or conventional flours had a similar glycaemic response when made from wholemeal or refined flours (28), which accords with previous studies that have compared the glycaemic index of breads made from wholemeal and refined wheat flours [31,32].

A range of different animal models have been used to assess starch digestibility in the upper gut but many have significant limitations and, importantly, these models have not been validated clinically. Granfeldt and colleagues [33] used the antibiotic-treated rat model to show that the RS content of corn bread was very high (32%). However, the results are questionable as Carvajal-Aldaz and colleagues [34] reported that antibiotic treatment of rats had variable effects on the activity of the large bowel microbiota and its capacity to ferment carbohydrates, and also gut hormone activity, which may influence digesta transit rate and starch digestion in the small bowel. Other studies used either the colectomised rat [15] or the serial/terminal slaughter/direct sampling of rat ileal digesta technique to measure upper gut starch digestibility of a few conventional cereal foods and diets [35,36]. All these studies showed that starch digestibility was close to 100%, regardless of the animal model or type of food/diet that was fed. Only the study by Roe et al. [35] directly compared digestibility data from the rat model with that from human ileostomates and concluded that the rat assimilated much more of the starch in a flaked barley food.

In contrast, the current study showed reasonable agreement between the IRM and human ileostomy models for determining RS. For bread made from conventional flour, starch digestibility was 0.75 g/100 g in both models, whereas for HAW bread it was 15% lower in the ileorectostomised rat. It is possible that the lower reported RS level for HAW was due to differences in digesta transit between the two models. Although we did not measure the transit rate, which is a major determinant of small intestinal starch digestibility, anatomical differences (ileorectostomised rats had intact rectal tissue) and a markedly lower digesta moisture content suggest that digesta transit was slower in the rat model. Delaying passage of food along the small bowel would allow more time for starch digestion and reduce the total amount of starch that is excreted from the upper gut. It is also possible that intestinal structural and functional differences between species [37] also contributed to small differences in starch digestibility between the models. However, it is unlikely that the relatively older age of the study participants impaired starch digestibility compared to the rats, as Table 1 shows that for the bread made from conventional wheat (LAW-R and LAW-W), >99% of starch was digested.

A methodological limitation of the rodent model is the high level of bread that had to be included in the diet (55% compared to 4–5% in the human diet). However, this requirement is unlikely to have influenced the capacity of the rat gut to assimilate starch. Indeed, starch digestibility was numerically greater in the rat model, suggesting that intestinal amylolytic capacity was not compromised. This is consistent with the fact that ileal starch output increases linearly with increasing starch intake but digestibility (output as a proportion of input) of a given type of starch remains constant across a wide range of intakes, at least in humans. Another difference between the rodent and human models is that the volunteers had well established ileostomies (>10 years) but it is unlikely that the different intervals between surgery and experimentation affected starch digestibility. In ileorectostomised rats, the indigestible starch content of breads was greater than in the flours. This is consistent with previous studies which showed that specific ingredients and baking conditions, such as the baking temperature and duration, can augment the RS content of bread [38,39]. The level of RS in bread made from refined flour is most likely due to starch retrogradation as the loaf cooled after baking.

The current study also showed that ileorectostomised rats fed the HAW-R flour diet had lower body weight gain during the initial 11-d feeding period compared to the LAW-R flour diet. As the intake of both diets was similar, it is likely that the reduced metabolizable energy of the HAW-R diet (higher in resistant starch) contributed to the lower growth rate. In addition, during phase 2 and 3 when body weight gain was similar between diets, diet intake was approximately 10% higher (although not significantly) for the HAW compared to the LAW group. Previous studies have shown that rats increase their food intake to compensate for dilution of dietary metabolizable energy [40], but other factors, namely increased large intestinal fermentation and subsequent changes in gut hormones were suggested as the main mechanisms responsible for the reduction in body weight and adiposity that was observed [40,41]. As the effect of HAW-R on lowered body weight gain was only observed during the initial phase when animals were still in their rapid growth stage, metabolic changes in response to HAW consumption during periods of increased weight gain are worth exploring further.

## 4. Materials and Methods

### 4.1. Human Study

#### 4.1.1. Study Population

Six individuals (four women, two men) with a mean age of 64 (range: 56–70) participated in the study. All study participants had well-established ileostomies having undergone minimal small-bowel resection (apparently <10 cm) for inflammatory bowel disease or cancer and all had conventional and well-functioning permanent ileostomy. They were in good health, without symptoms or signs of small intestinal inflammation or dysfunction. The exclusion criteria were: the use of any form of drug therapy, medication, or supplements on a regular basis that may interfere with bowel function, and the definite or suspected personal history of adverse events or intolerance of starchy or other foods, which may be tested in this study. Participants provided written, informed consent to the study protocol approved by the Commonwealth Scientific and Industrial Research Organisation (CSIRO) Human Research Ethics Committee. This study was registered at anzctr.org.au: ACTRN12620000898954.

#### 4.1.2. Recruitment and Screening

The participants were recruited from the CSIRO Nutrition and Health Research Clinic database. To compensate participants for time spent in the trial, participants were provided with gift vouchers on completion of the study to an amount equivalent to time spent in the study.

Participants were provided with information about the study design and, if interested, a first screening telephone questionnaire was administered to determine general eligibility. If eligible, participants were acquainted with the study procedures and all eligible individuals were invited to commence the study.

Study participants were recruited from 8 July 2014 until 11 August 2014 and 11 people were screened by telephone (Supplementary Materials Figure S1). As we were unable to recruit the intended eight study participants, a total of seven study participants were enrolled in the study. One person withdrew from the study before commencement as they were no longer available.

#### 4.1.3. Study Design and Intervention

The study was designed as a cross-over randomised trial based on the method we have described previously [26] and conducted at the study participant’s home and/or workplace. The trial was divided into two test periods, each of which consisted of four consecutive collection days (total of 8 days). There were a total of four treatments, and the treatment sequence was randomised through the use of a Latin square randomization sequence that included 4 unique sequences: ABDC, BCAD, CDBA, and DACB. Randomized allocation was conducted by the study manager who was also responsible for unblinding the data once statistical analysis had been completed by the project leader.

Participants and project staff were blinded to the composition of each test bread, which was designated by differently coloured labels. The test breads were only decoded once preliminary statistical analyses were completed. There were no discernible differences in taste, texture, or appearance for breads made from either HAW or conventional LAW flours. However, there were obvious differences between breads made from wholemeal and refined flour.

The four breads, low amylose wheat-refined (LAW-R), LAW-wholemeal (LAW-W), and high amylose wheat-refined (HAW-R) or HAW-wholemeal (HAW-W), were made from LAW or HAW flour according to a standard bread recipe as described previously [23]. The test breads were formulated and baked by the Australian Export Grains Innovation Centre (North Ryde, NSW, Australia) and stored frozen. Samples of each test bread were analysed for starch, RS, sugar, total dietary fibre, fat and protein content (Table 4). Freeze-dried and milled samples of each test bread were analysed in duplicate according to standard Association of the Official Analytical Chemists methods.

The study was run for four days (Monday to Thursday) over two consecutive weeks. During the two four-day treatment periods and for 24 h before each test period, the participants consumed a diet consisting of foods commonly consumed in a Western diet, which contained low levels of starch. On the first day of each four-day treatment period, participants consumed their assigned baseline foods only. For the subsequent three days, participants were randomly assigned to consuming 65 g of two different test breads at breakfast on two different days or continuing with the baseline diet (no bread). Study participants were provided with all the foods consumed during these two four-day periods of dietary restriction. The test breads were eaten by the participants in addition to the other foods consumed as part of the low-starch diet and a food diary was completed with any deviations recorded.

#### 4.1.4. Stoma Digesta Sample Collection

To minimize the bacterial degradation of the stoma effluent, the volunteers emptied their stoma bags every 2 h until 2100 on the test days, and the contents were placed in portable freezers (−20 °C). The final collection for each test starch was made at 0700 on the day after the starch was ingested so that the stoma were collected for 24 h after consumption. The stoma digesta samples from each volunteer collected during the 24 h after the ingestion of test breads were defrosted, pooled, homogenized, and subsampled for analysis.

#### 4.1.5. RS Analysis

The starch content of the stomal effluent was determined by a modified version of Association of Official Analytical Chemists method 996.11 [43] and all samples were analysed in triplicate.

In brief, freeze-dried digesta (100 mg) was suspended in 80% ethanol (5 mL) and the mixture was heated at 80 °C for 10 min. The mixture was centrifuged (2000× *g*, 10 min) and the supernatant was removed by aspiration. The residue was suspended in 2M potassium hydroxide (2 mL) and stirred at room temperature for 20 min after which time the solution was taken to pH 4.5 by addition of 30% *v*/*v* acetic acid (1.9 mL). Amyloglucosidase (0.1 mL, 330 U, Megazyme, Bray Ireland, Wicklow, Ireland) was added and the mixture heated at 50 °C for 60 min. The mixture was cooled and made up to 40 mL with water.

Glucose was determined using D-glucose assay kit (#K-GLUC, Megazyme Bray Ireland). An aliquot of the above solution (0.1 mL) was transferred to a 5 mL culture tube and GOPOD solution (3 mL) was added. The tube was heated at 50 °C for 20 min and the glucose concentration determined by absorbance at 510 nm against a glucose standard curve (0.06–1.0 mg/mL) on an Agilent Cary 100 spectrophotometer. The starch in the sample was determined by multiplying the total weight of glucose in the sample by 0.9 to convert the weight to starch. All reagents used were of analytical grade obtained through Merck Sydney Australia.

### 4.2. Animal Ileostomy

#### 4.2.1. Rats and Ileorectostomy Surgery

Five-week-old male Sprague-Dawley rats (*n* = 4) obtained from Animal Resource Centre (Perth, Australia) were housed individually in standard wire mesh-based cages in a room with controlled heating (23 ± 2 C) and lighting (lights on at 07:00–19:00 h). They were given 1 week to adapt to these conditions which included free access to water and a fibre-free semi-purified diet that contained 652.5 g of low amylose maize starch, 250 g of casein, 50 g of corn oil, 2.5 g of choline bitartate, 35 g of mineral mix (AIN-76 formulation) and 10 g of vitamin mix (AIN-76 formulation) per kg of diet. The animals were then deprived of food overnight and ileorectostomy surgery was conducted as described previously [14].

Postoperatively, the rats were not allowed food and water for the first 24 h and were then fed the control diet for 9–10 days. They received a daily intramuscular injection of antibiotics at surgery and for five days thereafter. In the two days following surgery, the rats lost 10–13 g (7%) of body weight, but from days 3 all rats gained weight at a constant rate (3–4 g/day). All aspects of animal care were under the oversight of the CSIRO South Australia Animal Ethics Committee, and the approved project number was 794-12/15.

#### 4.2.2. Experimental Protocol

Following recovery from surgery, rats (*n* = 4) weighing 167 ± 11 g were randomly assigned to consume two different diets containing LAW or HAW refined flours (Table 4) for a period of 11 days for each diet (total 22 days), followed by an additional period of consuming the two diets for a further 5 days (total 10 days). The diets were provided as a powder and were not heat treated. The order of diet allocation was randomly determined by throwing a coin and the technical staff were blinded to the flour type (LAW or HAW) with the treatments decoded at the end of the study by a staff member independent of the study. After the flour diet testing period one rat developed a blockage of the anastomosis site and was removed from the study. The remaining rats (*n* = 3) then consumed two additional diets that contained LAW-R and HAW-R bread (Table 4) for 7 days (total 14 days) as this enabled direct comparison with the human data for the same breads. Since there was no prior work to evaluate the utility of the ileorectomised rat for quantifying RS, for ethical and operational reasons only refined breads were investigated in the first instance. Testing was not subsequently extended to include wholemeal breads because the human ileostomy trial had shown that these and the refined breads contained essentially the same amount of RS.

Ileal effluent (faeces) was collected twice daily throughout the study and stored frozen. Faceal samples from each 24 h period (09:00 h) were freeze dried and milled prior to analysis of starch as described for the human ileostomy protocol.

Feed conversion efficiency was calculated as the ratio of body mass gain (g/day)/feed intake (g/day) for each feeding period.

### 4.3. Statistical Analysis

The sample size calculation estimated that six study participants were required to provide 80% chance of detecting a 200% increase in ileal starch excretion above baseline (*p* < 0.05) and allows for a potential withdrawal of two study participants. Basal starch excretion was estimated at 0.5 g and consumption of a standard serving portion of a cereal product (~60 g) made from HAW and containing 4% RS is expected to yield an additional 2.4 g of starch at the terminal ileum. Therefore, on the days when HAW is eaten, we anticipate that total starch recovery to be in the order of nearly 3 g (i.e., a four-fold increase over baseline). The RS content of the control product is estimated to be much lower (approximately 2%).

For the intervention in ileorectostomised rats, study power was calculated using starch digestibility data from an ileorectomised rat study, in which high amylose maize starch diets reduced starch digestibility by 34.4 ± 1.1% (25). Based on these values, with a target power of 80% and using a two-tailed *t*-test it is estimated that a sample size of at least n = 3 was required. No criteria was set to exclude animals during the experiment and all data collected was analysed.

The RS content of a given test food was calculated as the difference between ileal starch output for the test food and that of the control (i.e., low starch) diet. RS values are expressed on either a food or total starch basis. Values are expressed as means with standard deviation of four replicates for test diets and three replicates for test breads in rats and six replicates for each test bread in ileostomates. Data was checked for normal distribution and if not, data was log transformed if not prior to analysis. Differences between test foods in each model were assessed using a 2-tailed student’s paired *t*-test in Microsoft Excel and effects were considered significant at *p* < 0.05. Comparisons between the human and animal model were asessed using a two-tailed unpaired student *t*-test and percent change in RS was determined by the following formula; 100 × (human ileostomy model RS value-IRM RS value)/human ilesotomy model RS value). In the ileorectostomised rat model, as total starch ingested, starch collected in digesta and undigested starch were similar between the 11 days and subsequent five days testing periods for the LAW-R and HAW-R diets, as determined by a two-tailed student’s paired *t*-test, the data was averaged over a total of 16 days.

## 5. Conclusions

This study confirmed that the human ileostomy model is the most direct and accurate method for quantifying starch escaping digestion in the small intestine. Our study established that breads made with HAW flour contain 8-fold more RS than breads made from conventional wheat. Importantly, these higher levels of RS are similar regardless of whether bread is made from either wholemeal or refined flour. HAW can therefore be used to enrich the RS content of a wider variety of processed foods, such as noodle and baked products, which are typically low in RS but consumed broadly across the global population. For bread containing a high level of amylose, the ileorectostomised rat model underestimated RS content. Further research on other starchy foods is required to determine if the ileorectomised rat is a suitable alternative to the human ileostomy model for measuring RS. Furthermore, provided the IRM is calibrated against human data, it may have merit for investigating digestibility of starch in prototype foods and unprocessed products, such as flour. It could also be useful for studying RS formation (RS3, retrograded starch) during food preparation, manufacture and storage. The IRM showed that HAW slowed the body weight gain of rapidly growing rats independent of energy intake. This finding also warrants further investigation.

## Figures and Tables

**Table 1:** The effect of breads made from LAW and HAW flours on stoma digesta output and composition and starch digestibility in human ileostomates.

	LAW-R		HAW-R		LAW-W		HAW-W	
	Mean	SD	Mean	SD	Mean	SD	Mean	SD
Digesta wet weight (g/day)	548	129	542	97	629	254	693	174
Digesta moisture (%)	91.9	0.9	91.3	1.0	91.7	1.1	91.7	0.86
Digesta dry weight (g/day)	44	9	46	4	51	15	57 ^†^	11
Total starch ingested (g/day)	26.4	0.7	21.9 *	0.6	20.9	0.6	17.0 ^†^	0.34
Starch excreted (g/day)	0.8	0.2	4.5 *	0.2	0.65	0.2	3.9 ^†^	0.4
Undigested Starch (% of starch intake)	2.0	0.6	19.6 ^†^	1.0	2.0	0.5	21.1 ^†^	2.5
Resistant Starch (g per 100 g bread)	0.75	0.10	6.47 *	0.32	0.61	0.18	5.09 ^†^	0.61

LAW-R, low amylose wheat-refined flour; HAW-R, high amylose wheat-refined flour; LAW-W, low amylose wheat-wholemeal flour; HAW-W, high amylose wheat-wholemeal flour; SD, standard deviation. The digestibility of starches was calculated by using the following formula: digestibility {[(grams of ingested starch) (grams of starch output)]/(grams of ingested starch)} × 100. * Mean values were significantly different from LAW-R (*p* < 0.001). ^†^ Mean values were significantly different from LAW-W (*p* < 0.001).

**Table 2:** The effect of diets containing LAW and HAW bread and flour on starch ingestion and excretion in ileorectomised rats.

	Bread Diet				Flour Diet			
	LAW-R		HAW-R		LAW-R		HAW-R	
	Mean	SD	Mean	SD	Mean	SD	Mean	SD
Digesta wet weight (g/day)	3.0	0.7	7.3 ^††^	0.5	3.4	0.8	6.0 *	1.6
Digesta moisture (%)	53.2	1.0	49.0	4.4	52.9	5.8	49.7	3.6
Digesta dry weight (g/day)	1.4	0.3	3.7 ^†^	0.5	1.7	0.5	3.0 *	0.7
Total starch ingested (g/day)	6.9	0.3	7.7	0.5	8.0	1.1	8.1	0.4
Starch excreted (g/day)	0.12	0.03	1.24 ^††^	0.15	0.03	0.01	0.83 **	0.23
Undigested Starch (% of starch intake)	1.8	0.5	15.6 ^††^	2.0	0.8	0.4	11.2 **	1.6
Resistant Starch (g per 100 g)	0.76	0.22	5.48	0.76	0.4	0.1	10.3 ***	1.6

LAW-R, low amylose wheat-refined flour; HAW-R, high amylose wheat-refined flour. Test breads, *n =* 3. The digestibility of starches was calculated by using the following formula: digestibility {[(grams of ingested starch) (grams of starch output)]/(grams of ingested starch)} × 100. Mean values were significantly different from Bread LAW-R (^†^ *p* < 0.05, ^††^ *p* < 0.001). Mean values were significantly different from Test diet LAW-R (* *p* < 0.05, ** *p* < 0.001, *** *p* < 0.0001).

**Table 3:** Body weight gain, feed intake and feed conversion efficiency in ileorectomised rats.

	LAW-R		HAW-R	
	Mean	SD	Mean	SD
Test diets				
Phase 1, 11 day (*n* = 4)				
Body weight gain, g/day	6.2	1.7	4.0 *	1.6
Feed intake, g/day	21.2	1.0	22.1	3.7
Feed conversion efficiency	0.25	0.01	0.16	0.1
Phase 2, 5 day (*n* = 4)				
Body weight gain, g/day	2.1	0.6	1.8	1.1
Feed intake, g/day	20.9	2.2	22.1	1.1
Feed conversion efficiency	0.08	0.01	0.10	0.02
Bread				
Phase 3, 7 day (*n* = 3)				
Body weight gain, g/day	2.5	0.3	2.8	0.7
Feed intake, g/day	18.9	0.9	21.8	1.3
Feed conversion efficiency	0.13	0.01	0.13	0.05

LAW-R, low amylose wheat-refined flour; HAW-R, high amylose wheat-refined flour; SD, standard deviation. * Mean values were significantly different from LAW-R (*p* < 0.05).

**Table 4:** Ingredient analysed and calculated composition of rat intervention test diets (as fed).

	Flour		Bread	
	LAW	HAW	LAW	HAW
Ingredients, g/kg				
LAW refined flour	55.45			
HAW refined flour		55.45		
LAW refined flour bread			55.45	
HAW refined flour bread				55.45
Casein	19	19	19	19
Sucrose	10	10	10	10
Sunflower oil	7	7	7	7
Vitamin mix	1	1	1	1
Mineral mix	3.5	3.5	3.5	3.5
L-Cysteine	0.3	0.3	0.3	0.3
Choline	0.25	0.25	0.25	0.25
Alpha-cellulose	3.5	3.5	3.5	3.5
Total	100	100	100	100
Composition of diets				
Total starch	34.8	32.0	38.1	33.7
Protein	21.7	24.5	17.0	17.0
fat	9.4	9.5	7.8	8.0
Total dietary fibre	5.7	10.1	4.8	6.0

LAW, low amylose wheat (a blend of Sunstate and Chara wheat varieties); HAW, High amylose wheat. Bread was dried at 37 °C for 24 h prior to inclusion in the diet. Energy content of the diets was calculated by using Atwater coefficients and the macronutrient composition of the experimental diets. A value of 8 kJ/g was used as the coefficient for dietary fibre. Vitamin and mineral mixes were based on AIN-93 [42] formulation.

## Data Availability

The data presented in this study are available on request from the corresponding author.

## Supplementary Materials

The following are available online at https://www.mdpi.com/2072-6643/13/1/91/s1. Figure S1: Participant flow diagram.

## Abbreviations

The following abbreviations are used in this manuscript:

HAW-R	High amylose wheat-refined
HAW-W	High amylose wheat-wholemeal
IRM	Ileorectostomised rat model
LAW-R	Low amylose wheat-refined
LAW-W	Low amylose wheat-wholemeal
RS	Resistant starch
SCFA	Short-chain fatty acid
