# Supplementary figures and images for: Evaluation of an Ileorectostomised Rat Model for Resistant Starch Determination
Source: Nutrients. 2020 Dec 30;13(1):91. doi: 10.3390/nu13010091 (PMC7824714; doi:10.3390/nu13010091)

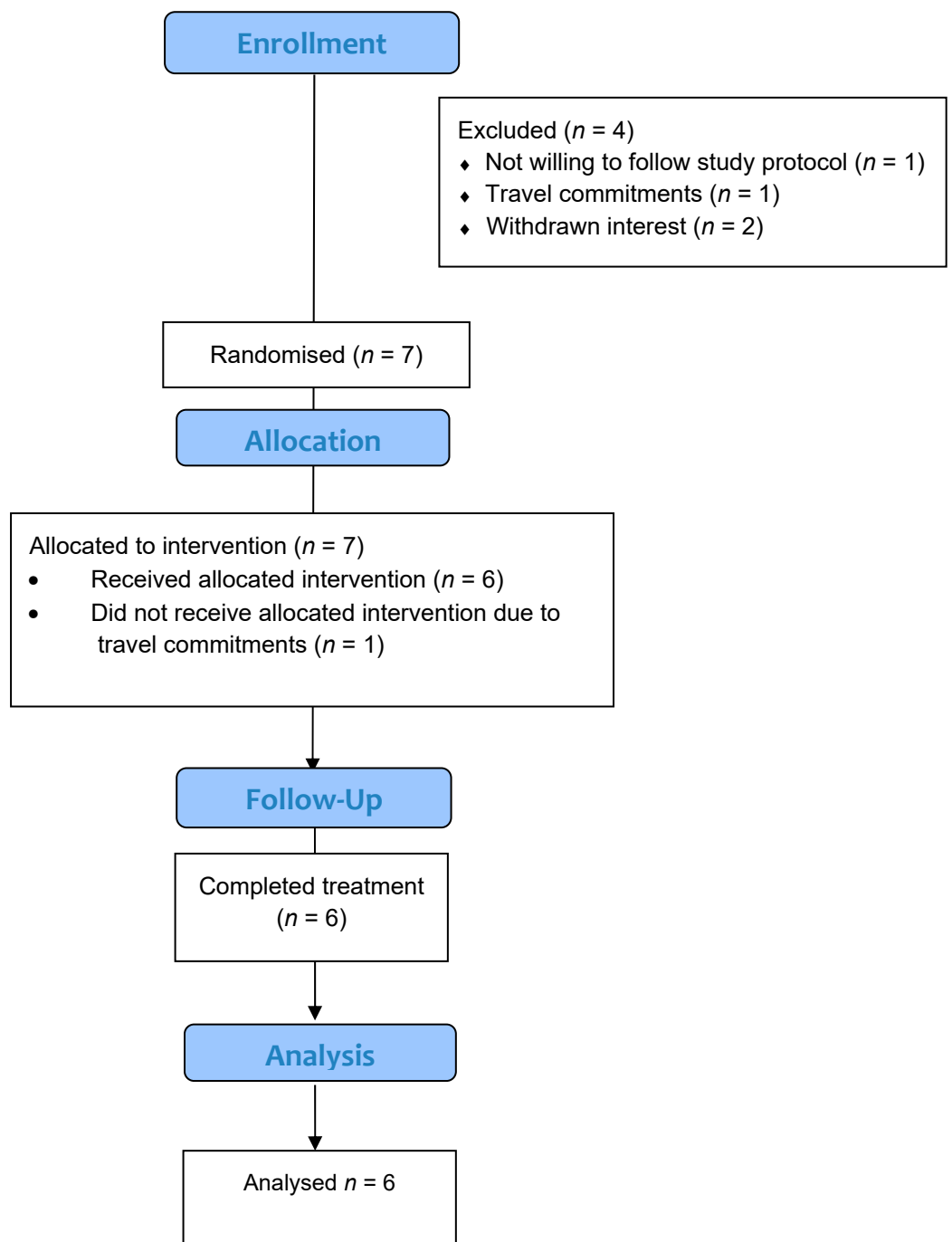

**Figure S1.** Participant flow diagram.

Supplement: Supplementary file 1 [file nutrients-13-00091-s001.pdf]
